# Union Status and Disability Pension

**DOI:** 10.1007/s10680-023-09670-7

**Published:** 2023-07-04

**Authors:** Solveig Glestad Christiansen, Øystein Kravdal

**Affiliations:** 1 Department of Alcohol, Tobacco and Drugs, Norwegian Institute of Public Health, PO Box 222, 0213 Skøyen, Oslo, Norway; 2 Centre for Fertility and Health, Norwegian Institute of Public Health, Oslo, Norway; 3 Department of Economics, University of Oslo, Oslo, Norway

**Keywords:** Union status, Cohabitation, Disability pension, Register data: Norway

## Abstract

A lot is known about the association between marital status and mortality, and some of these studies have included data on cohabitation. Studies on the association with health problems, rather than mortality, are often based on self-reported health outcomes, and results from these studies are mixed. As cohabitation is now widespread, more studies that include data on cohabitation are needed. We use Norwegian register data that include detailed information about union status and all cases of disability pensioning from 2005 to 2016. We employ Cox regression analysis and a within-family design in order to control for hard to measure childhood characteristics. Compared to the married, the cohabiting have a somewhat higher risk of receiving disability pension due to mental disorders, and for men also due to physical disorders. Receipt of disability pension is most common among the never married, especially for men. The association between union status and disability pensioning is stronger for mental than for physical disorders.

## Introduction

An extensive literature has documented that the married have a lower mortality than the unmarried. An elevated mortality has been reported among both the never married, the divorced and the widowed, and for all causes of death, bar some forms of cancer (Berntsen, 2011; Franke & Kulu, 2018). The mortality advantage of the married has also been increasing during the last decades (Berntsen, 2011; Kravdal et al., 2018).

Even if most common in young adulthood, cohabitation has become more prevalent also in higher age groups, especially in Northern European countries (Sánchez Gassen & Perelli-Harris, 2015). In spite of this increase in cohabitation, relatively few mortality studies have distinguished between the married and the cohabiting. These studies have usually indicated that the cohabiting have a somewhat higher mortality than the married but, in most cases, lower than those living alone (Drefahl, 2012; Koskinen et al., 2007; Staehelin et al., 2012).

Studies on the association between marital status and health have shown worse self-rated health among the non-married and particularly among the formerly married (Liu & Umberson, 2008). Among the studies that have also taken cohabitation into account, most of those from the US have found that the cohabiting have poorer self-rated health than the married (Denney et al., 2013; Fuller, 2010; Hsieh & Liu, 2019; Lamidi, 2020; Wang, 2022). In contrast, an investigation from Finland showed no association between cohabitation and self-rated health (Joutsenniemi et al., 2006a, 2006b), while another analysis showed no association except among cohabiting divorced women in the Netherlands (Joung et al., 1995). Perelli-Harris et al. (2018), who considered the US, UK, Australia, Germany and Norway, found worse self-rated health among the cohabiting than the married only in the US and UK.

Research on the relationship between marital status and mental health (rather than health more generally) has usually utilized self-reported data and has found that the never married and formerly married have poorer mental health than the married (Jang et al., 2009; Kessler et al., 2003; Yan et al., 2011). The research into differences in mental health between the cohabiting and the married has mainly reported no difference in mental health symptoms (Joutsenniemi et al., 2006a, 2006b; Reneflot & Mamelund, 2012; Ross, 1995), in episodes of major depression (Bulloch et al., 2017; Lindeman et al., 2000) or in psychotropic medication use (Reneflot & Mamelund, 2012; Van Hedel et al., 2018). A longitudinal analysis from Norway pointed in the same direction. It showed, for example, no change in the annual number of primary health care consultations because of mental disorders when a cohabiting couple married (Kravdal et al., 2022). However, a few studies have found evidence of more depressive symptoms among the cohabiting than the married (Brown, 2000; LaPierre, 2009; Marcussen, 2005; Yucel & Latshaw, 2022) at least among men (Brown et al., 2005).

Whereas the vast majority of the previous investigations of the association between union status and health related outcomes have focused either on mortality or self-rated physical or mental health, some have also dealt with work impairments. For example, some authors have reported an increased chance of sickness absence or of receiving disability pension around the time of divorce (Blekesaune & Barrett, 2005; Brüggmann, 2020; Eriksen et al., 1999; Kalmijn, 2005), although Couch et al. (2015) found an association only in the long run among men who did not remarry.

Samuelsson et al. (2012) compared the unmarried to the married and found that the unmarried had a higher risk of receiving disability pension and particularly disability pension due to mental disorders but did not distinguish between the never married and previously married. Unfortunately, studies that include data on cohabitation are lacking.

There are many reasons why union status may affect mortality, health or the probability of work impairment. For example, partners typically pool their (economic) resources and have advantages of scale, and they may benefit from mutual social support and from monitoring each other’s health behavior. The strength of these potential mechanisms may depend on the type of union, with marriage often being found to involve higher levels of commitment and relationship quality than cohabitation (Brown et al., 2017, 2022; Perelli-Harris et al., 2018). Having lived a large part of life alone, which is most common among the never married, has been linked to a number of health problems (Davidsen et al., 2022; Kriegbaum et al., 2009, 2011). Divorce and the underlying relationship problems may cause an immediate and possibly also longer-term deterioration of health (Kravdal & Wörn, 2023; Reneflot et al., 2020).

However, reported associations between union status and health related outcomes are also partly a result of selection effects. More specifically, the chance of entering or leaving a union, or of transitioning from cohabitation to marriage, is likely influenced to some extent by health and characteristics such as education, occupation, and personality. These factors may also impact health later in life as well as disability pensioning and mortality. Unfortunately, while it is often possible to control for some joint determinants of union status and health related outcomes, others may not be included in the data at hand or are very hard to measure.

The aim of this study is to investigate the association between union status and all cause disability pensioning, as well as disability pensioning due to mental or physical disorders. This is an expansion on earlier research which has mainly focused either on mortality or self-reported health measures. We improve upon earlier studies of disability pension—in two ways. First, we take cohabitation into account. Second, our access to nationwide register spanning multiple generations (itself an asset), makes it possible to estimate sibling fixed effects models. With such models, which have rarely been used in studies of the relationship between union status and health (and never in studies of disability pensioning), one controls for factors shared by siblings, such as genes (full siblings share approximately 50% of their genes) and environmental and family characteristics in childhood and early adulthood shared by siblings. With this kind of analysis, we contribute to broaden and improve the knowledge about the association between union status and health related outcomes, which may be valuable to health care personnel as well as those in charge of planning the future health services. We may also be considered as feeding into an intersecting research issue of great importance for the public, political and scholarly discussions of contemporary family behavior: how consensual unions and marriages differ.

## Methods

### Data

The data includes all individuals born in Norway between 1945 and 1975, who were alive and living in Norway at some point between 2005 and 2016 when aged between 40 and 61. Data on sex, year and month of birth, dates of death and migration (if any), as well as union status and municipality of residence at the beginning of each year were extracted from the Population Register. Information on the highest level of education was taken from the National Education Database. Data on year and month of being granted disability pension and the underlying diagnosis were provided by the Norwegian Labor and Welfare Administration. Data were linked across these administrative registers by means of the individual identifiers made available to us. These identifiers were (for data protection purposes) recoded versions of the personal identification numbers assigned to all individuals who have lived in Norway for some time after 1964.

Immigrants were excluded from the analysis because (compared to the Norwegian born) there is a higher chance that their *de jure* marital status is different from their de facto marital status. Since very few in these age groups have lost a spouse, we excluded the widowed from the analysis. The very small number of individuals with missing information about union status or region of residence were also excluded, as were those who were married but not living with their spouse, and those who were registered as cohabiting but where information about the partner was missing. The data owners and the Regional Committees for Medical and Health Research Ethics have approved the use of the data.

In Norway, disability pension may be granted if work capacity is reduced by (in most cases) at least 50% due to illness or injury. Applications for disability pension are determined by the Norwegian Labor and Welfare Administration based on recommendations from medical personnel. Disability pension is a permanent benefit with a compensation of around 66% of the income prior to disability. In the last years, around 20% of those granted disability pension have been in their forties and around 30% in their fifties (Norwgian Welfare Administration, 2022).

### Variables

The outcome variable was whether disability pension had been granted, and the time when this happened. In addition to analyzing all cause disability pensioning, we estimated models specifically for two broad subgroups: disability pensioning due to mental disorders (ICD-9: 290–319, ICD-10: F00–F99) or to physical disorders (all other ICD codes). Information about diagnosis was missing in about 5 percent of cases. The union status variable distinguishes between the married, the cohabiting, and those who live alone and who are either never married, or divorced or separated. The cohabiting may be either never married or divorced. Level of education was categorized into less than high school education, high school, and short and long tertiary education**.** In addition to including a county indicator in the analysis (there are 19 counties in Norway), we also included a measure of centrality categorized into six groups. The centrality index captures how many jobs and service functions that are located within a reasonable travel time from the place of residence (Høydahl, 2017). Year of birth (1-year groups) was included in all models.

### Model

The analytical sample was restricted to those with at least one same-sex full sibling who could be identified in the data and who was under exposure for the same transition in the study period. Cox proportional hazard regression models with age as the underlying timescale were estimated. Individuals were followed from age 40, January 1, 2005, or time of immigration, whichever came last. (Those who already had a disability pension at this time were, of course, not included in the analysis.) The end of follow-up was when they were granted disability pension, when reaching age 62 (when many can take early retirement), at the time of death or emigration, or the last date covered by the data (30th June 2016). The date of disability pensioning was set to the 15th of the month that disability pension was granted. Sex and year of birth were included as time invariant covariates, whereas union status, education, county of residence and centrality were included as time varying covariates. Periods of temporary absence abroad were excluded.

In some of the analysis, sibling fixed effects models (so-called “stratified” models) were estimated within the same-sex sibling groups. The motive was to find out whether there still is an effect of partnership on the disability pension transition when we control for all characteristics shared by same-sex siblings, not (given how difficult it is to compare across models) to identify how much of the originally estimated effect that is a result of such characteristics.

In the following, we will for simplicity refer to those who live alone and have never been married as “never married,” and those who live alone and are formally divorced or separated as “divorced.”

## Results

Characteristics of the sample at baseline and number of disability pensions granted during follow-up in various categories of union status, education, centrality and year of birth are shown in Table 1. There were 33,139 cases of disability pensioning among 437,718 men, while there were 42,411 cases among 371,400 women.

**Table 1:** Characteristics of the study population at baseline and number of disability pensions granted

	Men				Women			
	*n*	% of sample	Number of disability pensions	%	*n*	% of sample	Number of disability pensions	%
Total	437,718		33,139	7.8	371,400		42,411	11.4
*Education*^*a*^								
Less than high school	136,955	31.3	18,889	13.8	128,371	34.6	24,200	18.9
High school	165,649	37.8	10,680	6.1	99,613	26.8	9848	9.9
Short tertiary education	89,974	20.6	3245	3.6	119,215	32.1	7557	6.3
Long tertiary education	43,977	10.1	706	1.6	23,659	6.4	650	2.8
No or missing	1,163	0.3	231	20.0	542	0.2	156	28.8
*Union status*								
Married	67,899	58.1	15,210	11.8	225,423	60.7	23485	10.4
Never married	254,205	15.5	7981	6.0	41,629	11.2	5248	12.6
Divorced	40,208	9.2	5165	12.9	43,015	11.6	7282	16.9
Cohabiting	75,406	17.2	4783	6.3	61,333	16.5	6396	10.4
*Centrality*								
1 (most central)	61,998	14.2	2866	4.6	55,561	15.0	3734	6.7
2	98,967	22.6	6909	7.0	86,163	23.2	9162	10.6
3	116,859	26.7	8977	7.7	99,720	26.9	12,139	12.2
4	76,764	17.5	6915	9.0	64,018	17.2	8683	13.6
5	54,586	12.5	4549	8.3	43,912	11.8	5615	12.8
6 (least central)	28,544	6.5	2923	10.2	22,026	5.9	3078	14.0
*Year of birth*								
1945–1949	35,257	8.1	3570	10.1	19,027	5.1	2500	13.1
1950–1954	63,915	14.6	8338	13.1	48,245	13.0	8572	17.8
1955–1959	82,246	18.8	8640	10.5	70,942	19.1	11,240	15.8
1960–1964	91,118	20.8	6361	7.0	81,653	22.0	9570	11.7
1965–1969	92,305	21.1	4528	4.9	84,296	22.7	7499	8.9
1970–1975	72,877	16.7	1702	2.3	67,237	18.1	3030	4.5

^a^The “less than high school” category includes all those who had not completed upper secondary education regardless of whether they had finished only primary school (older cohorts) or lower secondary school (later cohorts). The “High School” category includes those who had completed upper secondary education. “Short tertiary education” includes those who have attained an educational level reckoned as corresponding to 1–4 years of tertiary schooling, while the last group includes those with even more schooling

^b^Those in civil partnership are combined with the married, whereas those formerly in a civil partnership are combined with the divorced

Table 2 shows the association between union status and all cause disability pensioning. In the regular Cox models (without sibling fixed effects), (Model 1), the never married, divorced and cohabiting have a higher risk of receiving disability pension than their married counterparts among both men and women. Among men, being never married is associated with the highest excess risk, whereas among women there is no difference between the never married and the divorced. Among both sexes, the excess risk compared to the married is smallest among the cohabiting. Results from the sibling fixed effects models (Model 2) confirmed the pattern from the regular models. For the never married and the cohabiting, the estimates hardly changed, but for the divorced, the excess risk is reduced, particularly for women, so that according to the sibling fixed effects model estimates, being never married is associated with the highest risk also among women.

**Table 2:** Hazard ratios with 95% confidence intervals for the association between union status and all cause disability pension, men and women aged 40–61 years, Norway, 2005–2016

	Men		Women	
	Model 1	Model 2	Model 1	Model 2
*Union status*				
Married	1	1	1	1
Never married	2.52 (2.45–2.59)	2.50 (2.38–2.64)	1.56 (1.51–1.61)	1.51 (1.43–1.59)
Divorced	2.06 (2.00–2.12)	1.88 (1.78–1.98)	1.53 (1.49–1.57)	1.35 (1.30–1.41)
Cohabiting	1.23 (1.18–1.27)	1.19 (1.13–1.26)	1.12 (1.09–1.15)	1.10 (1.05–1.15)

Model 1: Adjusted for birth cohort, education, region of residence and centrality

Model 2: Model 1 + sibling fixed effects

Table 3 displays the associations between union status and disability pensioning due to mental disorders. Results from the regular Cox models (Model 1) show that there are strong associations between being either never married or divorced and disability pensioning due to mental disorders, especially among men. The highest excess risk is found among the never married. The cohabiting also have a heightened risk, but this is smaller in magnitude. The results from the sibling fixed effects models (Model 2) confirm this pattern.

**Table 3:** Hazard ratios with 95% confidence intervals for the association between union status and disability pension due to mental disorders, men and women aged 40–61 years, Norway, 2005–2016

	Men		Women	
	Model 1	Model 2	Model 1	Model 2
*Union status*				
Married	1	1	1	1
Never married	6.51 (6.16–6.89)	6.37 (5.71–7.10)	3.23 (3.06–3.41)	3.02 (2.73–3.33)
Divorced	4.59 (4.32–4.88)	4.20 (3.77–4.69)	2.97 (2.82–3.12)	2.57 (2.36–2.80)
Cohabiting	1.62 (1.50–1.75)	1.68 (1.49–1.90)	1.49 (1.40–1.59)	1.47 (1.33–1.62)

Model 1: Adjusted for birth cohort, education, region of residence and centrality

Model 2: Model 1 + sibling fixed effects

The estimated association between union status and disability pensioning due to physical health problems are presented in Table 4. In the regular Cox models (Model 1), never married men display the highest excess risk of receiving disability pension compared to the married. Among women, the divorced display a higher excess risk than the never married. Among both men and women, the cohabiting also have a somewhat elevated risk. Including sibling fixed effects (Model 2) reduce the estimates for the divorced, and according to these models the highest excess risk among men is found for the never married, whereas the estimates for never married and divorced women are of comparable magnitude. In these models, cohabiting men still have a small excess risk, whereas there is no longer a significant disadvantage for cohabiting women.

**Table 4:** Hazard ratios with 95% confidence intervals for the association between union status and disability pension due to physical disorders, men and women aged 40–61 years, Norway, 2005–2016

	Men		Women	
	Model 1	Model 2	Model 1	Model 2
*Union status*				
Married	1	1	1	1
Never married	1.69 (1.62–1.75)	1.71 (1.60–1.82)	1.15 (1.11–1.20)	1.13 (1.06–1.21)
Divorced	1.57 (1.51–1.63)	1.42 (1.33–1.51)	1.20 (1.16–1.24)	1.08 (1.03–1.14)
Cohabiting	1.17 (1.13–1.22)	1.11 (1.05–1.19)	1.04 (1.00–1.07)	1.02 (0.97–1.08)

Model 1: Adjusted for birth cohort, education, region of residence and centrality

Model 2: Model 1 + sibling fixed effects

The heightened risks of receiving disability pension due to physical disorders among the non-married are of smaller magnitudes than the corresponding excess risks of receiving disability pension due to mental disorders. Across models, hazard ratios are also generally larger for men than for women. Additionally, the never married have a more clearly increased risk of disability pension compared to the divorced among men than women.

Note that the conclusion about the difference between mental and physical disorders, and between women and men, is based on hazard ratios. A somewhat different picture of the sex differences appears when we turn from a relative to an absolute scale. For example, at age 50 in 2010, the rate of transition into disability pension due to mental disorders—averaged over union status—is 0.004 per person year for women and 0.002 for men (own calculations from the data). Thus, even if the HRs for, for example, divorced men are much higher than for divorced women (4.6 vs 3.0), the absolute difference in the transition rate between the divorced and married may be larger for women.

The comparison between mental and physical disorders, however, holds also on an absolute scale. More specifically, the transition rates into disability pension due to physical disorders are 0.009 for women and 0.007 for men. These numbers are about twice as high as the corresponding numbers for mental disorders, but since the HRs are much less than half as large as for the mental diseases, the conclusion is that union status is more closely linked to disability for mental than for physical disorders also on an absolute scale—not only the relative scale.

## Discussion

The association between marital status and different health outcomes has been extensively studied. However, most of these studies have not included cohabitation, and those that have done so have largely dealt with mortality or self-reported physical and mental health outcomes. We expand on this research by using high quality register data covering all disability pensions granted in Norway from 2005 to 2016. We find that the never married and the divorced have the highest risk of disability pensioning (with the never married at a level above the divorced) and that the cohabiting in most cases also have a heightened risk compared to the married, albeit less markedly so. The excess risks among the non-married are, on the whole, larger for men than for women, and larger for disability pensioning due to mental than physical disorders.

### Benefits of Marriage

The potential causal mechanisms linking marriage to better health may include pooling of resources and economies of scale (Lersch, 2017; Zagorsky, 2005). A spouse is typically also an important provider of social support (Ross, 1995). In addition, a spouse may monitor health behavior and promote healthier habits in their partner (Umberson, 1992). Among those who have already been diagnosed with an illness, being married may increase the adherence with medical treatment (DiMatteo, 2004).

It is possible that men benefit more from marriage than women. Indeed, most studies of the association between marital status and mortality point in that direction (Rendall et al., 2011; Shor et al., 2012; Staehelin et al., 2012). One reason for a special marriage advantage for men may be that men are more satisfied with the social support they receive from their spouse than women (Xu & Burleson, 2001). Moreover, having a partner may be a particularly important buffer against loneliness for men (Peters & Liefbroer, 1997), and wives often exert more social control over their husbands than the other way round (Umberson, 1992).

### Cohabitation Versus Marriage

In contrast to our results, which show that cohabitation is associated with a higher risk of receiving disability pension due to mental disorders and for men also due to physical disorders, Drefahl (2012) found a heightened mortality risk only among cohabiting women. Although cohabiting and married individuals in principle should gain the same type of advantages from living with a partner, research has pointed to a number of differences between cohabiting and marital couples that might also impact the health benefits obtained. For example, cohabiters have been found to pool their economic resources to a lesser degree (Hamplova & Le Bourdais, 2009; Hamplová et al., 2014; Lyngstad et al., 2011). A number of studies have also revealed that cohabiting couples report lower relationship quality than the married (Aarskaug Wiik et al., 2012; Brown et al., 2017, 2022) and lower levels of commitment (Perelli-Harris et al., 2014; Stanley et al., 2004), although the difference between marriage and cohabitation may be smaller in societies where cohabitation is widespread (Aarskaug Wiik et al., 2012). As a result, the levels of social support and social control might not be as high as in marriages. In line with this, studies have shown that the cohabiting are more likely to have a problematic alcohol use and to smoke (Fuller, 2010; Li et al., 2010; Reneflot & Mamelund, 2012), or that they are less likely to attend preventative health care services (Blumberg et al., 2012).

However, the observed differences in disability pension between cohabiting and married individuals may also be partly a result of health or health determinants being important for the choice of union type. For example, Wiik and Dommermuth (2014) found that long-term or chronic health problems in young adulthood were associated with a lower probability of having entered marriage by age 40, but not with having cohabitation experience. Also, studies have shown that substance use is associated with a higher likelihood of being a cohabiter rather than married in young adulthood (Joy Jang et al., 2018), and that cohabiters with a history of depression are less likely to marry (Sandberg-Thoma & Kamp Dush, 2014). Furthermore, cohabitation has been linked to low education or low income and to earlier experience of union dissolution (De Jong Gierveld, 2004; Kravdal, 1999; Sassler & Lichter, 2020), which may also have adverse effects on later health.

### Comparing the Never Married and the Divorced

Our finding that there is a particularly high risk of disability pensioning among the never married, especially among men, is in line with some (Koskinen et al., 2007) but not all studies (Staehelin et al., 2012) on all- cause mortality using comparable groups. In contrast, in most studies of self-rated health, the conclusion has been that the divorced fare worse than the never married or that there is no difference between the two groups (Averett et al., 2013; Hsieh & Liu, 2019; Joung et al., 1995; Joutsenniemi et al., 2006a, 2006b; Lamidi, 2020). Studies of depression have also found no difference or higher risk of depression among the divorced than the never married (Bulloch et al., 2017; Reneflot & Mamelund, 2012). In support of our findings, some studies have reported that those who have spent a larger number of years of their adult life living without a partner–as is typically the case for the never married unless they have been in long-lasting consensual unions—have a higher mortality and a more problematic alcohol use (Kriegbaum et al., 2009, 2011). For men, an association between years living alone and inflammatory markers has also been found (Davidsen et al., 2022).

### Divorce

The excess risk of disability pensioning among the divorced, and particularly divorced men, is quite reasonable in light of the existing literature. Divorce is a stressful event which might cause mental and psychosomatic disorders, either in the short term or more permanently. While some studies have shown that mental distress increases during the last years preceding the partnership dissolution and declines after the break-up (Blekesaune, 2008; Ding et al., 2021; Kravdal & Wörn, 2023; Leopold, 2018; Van Scheppingen & Leopold, 2020), other studies have indicated more long term adverse health consequences of divorce on measures such as contact with primary healthcare services for mental health problems (Reneflot et al., 2020), mental distress (Mastekaasa, 1995), immune functioning (Kiecolt-Glaser et al., 1987) and physical illness (Kravdal & Wörn, 2023; Lorenz et al., 2006).

Relationships between divorce and health are likely to also reflect health selection or other types of selection. For example, in their study of 12 countries across the world, Breslau et al. (2011) found most mental disorders (including depression, anxiety disorders and substance abuse) to be associated with higher likelihood of divorce. In line with our findings, the evidence for a link between poor physical health and subsequent divorce is weaker. Bünnings et al. (2021) showed that a sudden deterioration in the mental health of either partner led to a doubling of the risk of separation, but no increasing in the risk of separation following a sudden deterioration in physical health. Syse and Kravdal (2007) reported no increased divorce rates following a cancer diagnosis, except among those diagnosed with testicular or cervical cancers, and Joung et al. (1998) found a heightened risk of divorce only among those who reported having multiple subjective health complaints or chronic conditions. Research into sex differences in the link between health and later divorce has shown diverging results (Karraker & Latham, 2015; Lam et al., 2020; Metsa-Simola et al., 2021; Monden & Uunk, 2013; Teachman, 2010).

### Mental and Physical Disorders

We detect larger associations for disability pensioning due to mental than physical disorders. This is in line with findings from earlier research which suggest that mental health is particularly closely associated with union status. For example, a number of longitudinal studies have shown that entering a cohabiting relationship or marriage is linked to an improvement in mental health (Chen & van Ours, 2018; Musick & Bumpass, 2012; Rapp & Stauder, 2020), although most of the improvement may take place in the years preceding the relationship (Kravdal et al., 2022). In addition, most mental disorders, such as anxiety, mood and substance use disorders are associated with a lower likelihood of marriage (Breslau et al., 2011).

The picture is more mixed when it comes to physical health and its risk factors. Marriage has been consistently linked to decreases in alcohol consumption and particularly harmful patterns of drinking such as binge drinking (Dinescu et al., 2016; Duncan et al., 2006; Kendler et al., 2016). However, less consistent results have been reported for smoking, with some researchers finding a decrease in smoking following marriage (Franks et al., 2002), while others do not (Duncan et al., 2006). With regard to physical activity, a number of studies have found a reduction following marriage (Rapp & Schneider, 2013; Werneck et al., 2020). Likewise, an association between marriage and an increase in BMI and the risk of being overweight has been reported (Mata et al., 2018; Werneck et al., 2020). Regarding health selection some have found that those who have poorer general health in young adulthood are less likely to marry (Lipowicz, 2014; Mr & Sloggett, 1998). Others have found little evidence for health selection into marriage (Fu & Goldman, 1996; Joung et al., 1998; Waldron et al., 1996). In the opposite direction, Syse (2008) found an elevated marriage probability among male cancer survivors.

### Other Confounders

The estimated relationships reflect not only casual effects of union status on disability pensioning, or the influence of earlier health (as just discussed in some detail), but also joint determinants that have not been controlled for in the model. Some of these are taken into account in the sibling analysis. However, inclusion of sibling fixed effects primarily reduces the estimates for the association between being divorced and disability pensioning. For the other union status groups, the estimates hardly change. This suggests that genes or other characteristics shared between siblings affect the likelihood of divorce and disability pensioning in the same direction, but that these factors otherwise do not contribute much to a relationship between union status and disability pension.

### Strengths and Limitations

The main strength of this study is the use of longitudinal register data that include information on not only marital status, but also on cohabitation, and where information about close family members makes it possible to apply a within-family design.

However, although sibling models control for shared childhood environment and shared genes, there might still be selection on characteristics that vary between siblings such as differences in health relatively early in life, socioeconomic resources acquired in adolescence or early adulthood, and personality.

Some studies of the association between union status and health have controlled for constant unobserved individual characteristics in an individual fixed effects analysis, but that requires repeated measurement of health outcomes (such as the annual number of consultations with general practitioners, as in the studies by Kravdal and Wörn (2023) and Kravdal et al. (2022)). Another approach could be to control even better than we have done for genetic selection and family environment by employing information on twins (not adequately available in our data). For example, Horn et al. (2013) found in such an analysis that the association between physical health and marriage among young adults was entirely due to non-random selection, whereas for mental health, there were benefits of being in a cohabiting or married relationship on depression, suicidal ideation and alcohol use.

A particularly important limitation of our analysis is that the outcome measure reflects a combination of health and, for a given health status, the ability to stay in work. The latter may depend on, for example, occupation and the willingness of employers to accommodate special needs. Another concern is that a focus on disability pension only captures relatively severe cases of mental and physical disorders. Furthermore, our conditioning on survival contributes, in principle, to a relationship between union status and disability pensioning because both these factors affect survival (i.e., there is a potential collider bias).

Finally, one should keep in mind that Norway has one of the highest recipiency rates of disability pension in the OECD and one of the most generous disability pension systems (OECD, 2009). The results from this study may not be directly transferable to countries with different welfare policies. Since disability pensioning is relatively common in Norway, perhaps, the associations between social characteristics and disability pensioning are less strong than in countries where disability pensioning is less prevalent.

### Conclusion

To conclude, this study—based on high quality register data—showed that those living alone, and particularly the never married, have a higher risk of receiving disability pension than the married. Cohabitation is also associated with a heightened risk of disability pensioning, but this is smaller in magnitude. These associations, except the link between cohabitation and disability pension due to physical disorders for women, hold after controlling for shared genes and childhood family environment using sibling fixed effect models. The associations are generally strongest for disability pensioning due to mental disorders and stronger for men than for women.
